# The abundance of gut bacterial families as an early marker of diet-induced metabolic shift: a study in young pre-obese rats and a hint to a potential prophylactic window

**DOI:** 10.3389/fphar.2026.1923730

**Published:** 2026-09-21

**Authors:** Johana Eyzaguirre-Velásquez, Alejandra López-Aguilera, Jorge Escobar-Luna, Claudia Ibacache-Quiroga, Javier A. Bravo, Marcela Julio-Pieper

**Affiliations:** 1 Grupo de NeuroGastroBioquímica, Instituto de Química, Facultad de Ciencias, Pontificia Universidad Católica de Valparaíso, Valparaíso, Chile; 2 Centro de Micro-Bioinnovación (CMBi), Escuela de Nutrición y Dietética, Facultad de Farmacia, Universidad de Valparaíso, Valparaíso, Chile; 3 Center for Interdisciplinary Research in Biomedicine, Biotechnology and Wellbeing (CID3B), Pontificia Universidad Católica de Valparaíso, Valparaíso, Chile

**Keywords:** childhood-adolescence, diet induced obesity (DIO), enteric nervous system, microbiota transfer, pre-obesity

## Abstract

The occurrence of overweight-obesity in youth has increased globally, with growing evidence supporting the relevance of the gut microenvironment as a pathophysiological factor. The time preceding obesity consolidation is particularly relevant to therapeutic management; therefore, we evaluated gut microbiota and inflammatory features in submucosal neurons during a status of diet-induced dysmetabolism prior to obesity. Rats received high-fat (HF) diet (62% calories from fat) from postnatal day 30 (p30) onwards, reaching moderate obesity by p90. At p45, although not overweight, they showed increased fasting glycaemia; between p45-60 they displayed higher adiposity and liver lipid content than rats fed chow (14% calories from fat); however, inflammation-related peptides remained unchanged in colonic submucosal neurons. Next, we investigated the role of microbiota in HF diet-induced metabolic alterations associated with that pre-obese status: caecal microbiota taken from p60 HF-rats was transferred to healthy recipients (orally, p30-45). Controls received vehicle or heat-inactivated HF microbiota. Microbiota transplantation from pre-obese rats increased glycaemia in healthy receptors, compared to those given heat-inactivated material. Next, we broadly characterized bacterial families from HF and control rats (p30-90) using Illumina MiSeq platform to sequence DNA from caecal content. In HF rats, Tannerellaceae and Bacteroidaceae abundances increased early (p45), whereas Muribaculaceae and Lactobacillaceae abundances significantly decreased at p60 and p90, respectively. Pre-obese caecal microbiota induced an increase in glycaemia in healthy rats, possibly contributing to pathology. It may also be an early metabolic shift marker. Characterization at the genus level may provide novel markers for obesity risk, improving the chances for successful interventions.

## 1 Introduction

The worldwide prevalence of overweight and obesity in children and adolescents has been steadily increasing over the past 30 years. In 1990, less than 8% of kids between 5 and 19 years of age displayed a BMI greater than 1 standard deviation above the median, whereas by 2022, 19.77% of kids were within this group (WHO 2025 (WHO, 2025)). Worryingly, 55% of obese children and 80% of obese adolescents will still carry this condition as they grow older (Simmonds et al., 2016), increasing the risk for cardiovascular disease, metabolic alterations and cancer in adulthood (Weihrauch-Bluher et al., 2019).

Growing evidence supports the relevance of the gut microenvironment as a pathophysiological factor to obesity. Moreover, it has been proposed that, together with an unbalanced, high fat/high sugar diet, the presence of an “unhealthy” gut microbiome may predict the development of obesity in kids (Rampelli et al., 2018). The time preceding the establishment of obesity is particularly relevant to therapeutic management. As such, early onset markers are valuable. This investigation set to establish very early signs of pre-obesity and to identify potential associated bacterial profiles. In order to do so, we used a high-fat diet-induced model of obesity in rats, and we focused in the juvenile period, when early changes in the submucosal enteric nervous system and in the luminal microbial contents of the large intestine were evaluated in parallel.

## 2 Methods

### 2.1 Animals

Juvenile male Sprague Dawley rats (6–9 week old, 150–250g) were used in this study. Rats were acquired from the animal facility at Facultad de Ciencias Químicas y Farmacéuticas, Universidad de Chile, and housed in the animal holding unit at Escuela de Kinesiología, Facultad de Ciencias, Pontificia Universidad Católica de Valparaíso (PUCV). They were kept under standard conditions of light and temperature, with food and water *ad libitum*. The animal model and experimental design was approved by the Ethics Committee at PUCV. Prior to tissue retrieval, rats were sedated by isoflurane inhalation and killed by decapitation, a method that follows the Directive 2010/63/EU of the European Parliament and of the Council of 22 September 2010 on the protection of animals used for scientific purposes.

### 2.2 Experimental design

Rats were weaned at p21 and HF diet was provided from p30 onwards. A first cohort of HF rats was used to monitor the pre-obese to obese transition by recording body weight, glycaemia and perigonadal fat mass at p30, p45, p60 and p90. Caecal content, liver and colon tissues were also extracted from this cohort and used for microbiota characterization, lipid extraction and immunofluorescence studies, respectively. Age-matched control rats were fed regular chow. A second cohort of HF rats were used only at p60 as caecal content donors for the microbiota transfer study.

Recipients were healthy, regular chow fed rats that were pre-treated with antibiotics (p27 to p30). Recipient rats received donor microbiota orally from p30 to p45; this group was named Cae. The microbiota transfer experiment included two control groups: these were age-matched recipients given either vehicle or fed with heat-inactivated microbiota from HF donors, generating groups Veh and HK, respectively.

### 2.3 Diet specifications

High-fat (HF) diet had an energy density of 5.1 kcal/g (Diet induced obesity rodent purified diet from TestDiet) and calories were distributed as follows: protein 18.1%, fat 61.6% and carbohydrates 20.3%. Control diet was a standard grain-based diet with an energy density of 4.1 kcal/g (ProLab RMH3000 from Purina). kilocalories in the control diet are distributed as follows: protein 25.967%, fat 14.022% and carbohydrates 60.011%.

### 2.4 Caecal microbiota transfer

Recipient rats were pre-treated with an antimicrobial cocktail to decrease endogenous gut microbiota. The cocktail contained wide-spectrum, nonabsorbable antibiotics neomycin, bacitracin, and vancomycin (100 mg/kg each) plus the antimycotic pimaricin (5 mg/kg) and was administered p.o. daily from p27 to p30.

Caecal material was obtained from HF rats at p60. Briefly, the caecum was removed and its content expressed through a small opening into microcentrifugue tubes. This material was divided into 0.25 mL aliquots, flash frozen in liquid nitrogen and stored at −80 °C until used. On the day of transplantation, three aliquots obtained from three different donors were thawed and individually processed in the following way: 0.2 mL of saline (0.9% NaCl) were added, then caecal material and saline were mixed by using a plastic pestle and the mixture was vortexed. This mixture was centrifuged and 0.2 mL from the supernatant was collected. The procedure was repeated now adding only 0.1 mL of saline, mixing, vortexing, centrifuging and collecting 0.1 mL supernatant. The supernatants obtained from three different donors (0.9 mL in total) were mixed and administered p.o. to one recipient rat. This procedure was repeated for each receptor rat daily from p30 to p45, to constitute group Cae. At p45, animals were killed to obtain peripheral blood, liver and perigonadal fat. Similar procedure was followed for both control groups: antimicrobial cocktail-treated rats that received either a daily dose of saline vehicle (Veh) or heat-inactivated caecal material (HK, 5 min at 100 °C), p.o. from p30-p45.

### 2.5 Bacterial family evaluation

Caecal material was obtained at each time point (p45, p60 and p90) for both control diet and HF diet rats. Also a start point group (p30) was included. This material was stored at −80 °C until used. Caecal material was homogenized using a bead beater mill (Bioprep 24, Hangzhou Allsheng Instruments, Zhejiang, P.R. China) with two cycles of 5.5 movements per second during 1 minute. Bacterial DNA was isolated using the QIAamp Fast DNA Stool Mini Kit (Qiagen) and the V3-V4 region of 16S rRNA gene was sequenced using an Illumina MiSeq platform with a pair-end protocol (2 × 250 bp; FISABIO sequencing services, Valencia, Spain).

Sequence quality was analyzed using Fasta QC (v. 0.11.5). Metagenomic analyses included identification of 16S rRNA codifying sequences, generation of operational taxonomic unit (OTU) and phylotyping (Truong et al., 2015; Schmieder and Edwards, 2011). Taxonomic profiles of bacterial communities present in the caecal samples were determined using MetaPhlAn2 software (v. 2.0).

### 2.6 Staining of enteric neurons

Enteric neuron morphometry as well as characterization of neuronal phenotype was performed by immunofluorescence. Rat colon tissue samples were rinsed with saline and fixed overnight in Zamboni’s fixative. Mucosa and muscle layers were stripped away and submucosal plexus preparations were processed as described before (Collins et al., 2024). Briefly, tissues were permeabilized in PBS plus Triton X-100, blocked in donkey serum and double labelled against HuC/D (pan-neuronal marker) plus one of the following antigens: Somatostatin (SOM), Substance P (SP), Vasoactive Intestinal peptide (VIP) and Neuropeptide Y (NPY). Immunostaining was visualized using appropriate fluorescently labelled secondary antibodies. For morphometrical analyses, microphotographs were taken and analysed with MultiGauge software (Fujifilm, Tokyo, Japan).

### 2.7 Statistical analysis

Data were analysed using the statistical software GraphPad Prism 8 (GraphPad Software, San Diego, CA, United States of America) and were expressed as mean ± SEM. One-way ANOVA with Dunn’s multiple comparisons test, or two-way ANOVA with Bonferroni post-test were used where appropriate. Differences showing a p-value of less than 0.05 were considered statistically significant.

## 3 Results

Whereas both control and HF rats gained weight as they grew, the experimental diet did not have an immediate effect on body weight increase. Based on Bagnol et al. (2012), a 110% threshold was defined to use the term “obese” (using the body mass of age-matched controls as reference) and in the present study, this was significantly reached only by p90 (see Figure 1A). However, fasting glycaemia was transiently increased in HF rats at p45 (Figure 1B). Also, epididymal fat and liver adiposity were significantly higher from p45 onwards and at p60, respectively (see Figures 1C,D). At p90 the liver of HF rats consistently showed a discoloration (see Figure 1E). The next analyses were performed in pre-obese rats, i.e., younger than p90.

**FIGURE 1:**
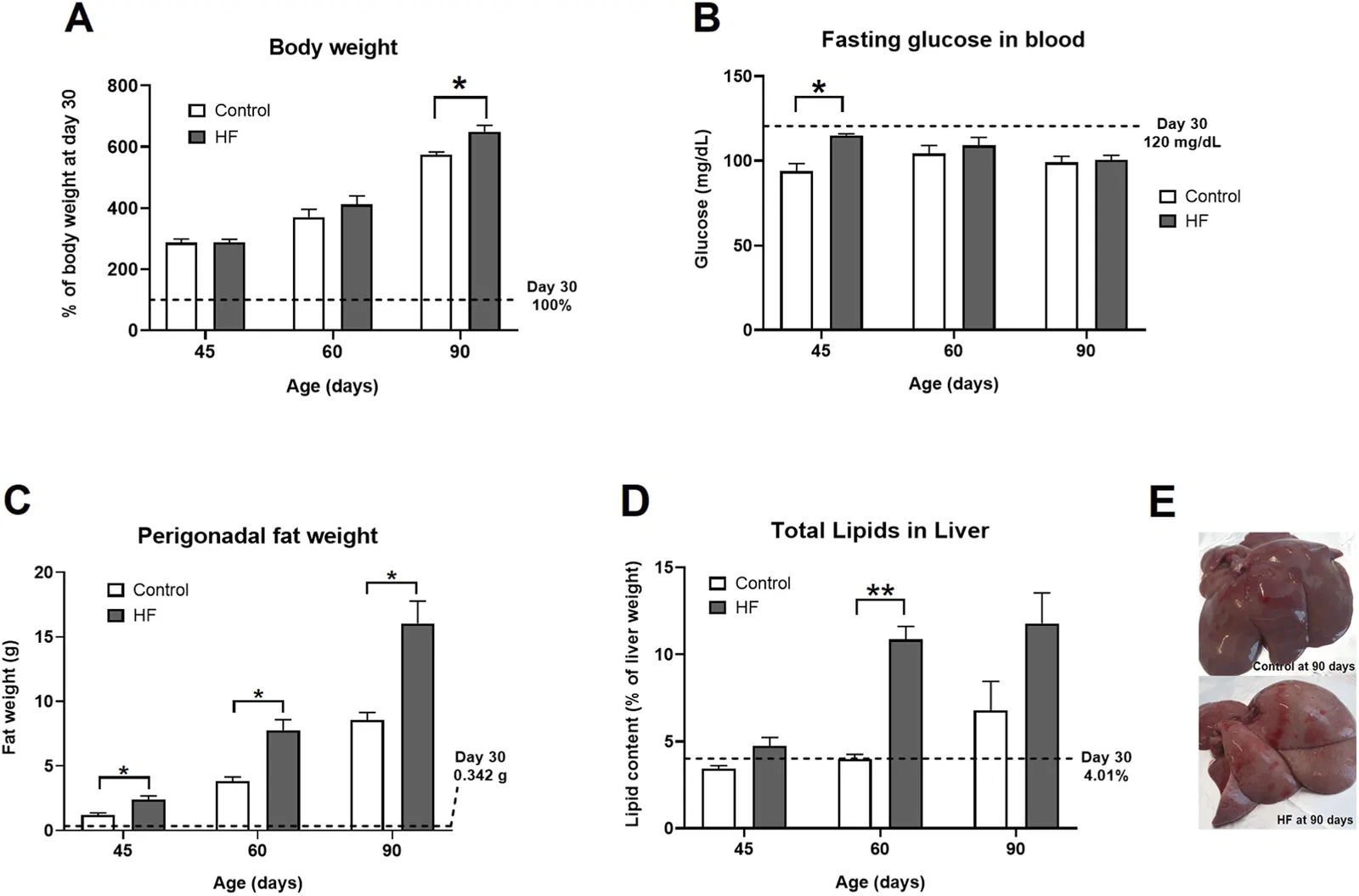
Anatomic and metabolic parameters in rats receiving HF diet. Body weight **(A)** was calculated as 100x((final weight–initial weight)/final weight). At p90, HF weight was 13.24% higher than controls. Blood glucose **(B)**, perigonadal fat weight **(C)** and liver lipid content **(D)** were obtained to represent metabolic status. Liver photographs **(E)** were captured immediately post-mortem. n = 5–6, *p < 0.05, **p < 0.01, two-way ANOVA, post‐test Bonferroni.

Colon submucosal neurons were identified using pan-neuronal marker HuC/D. In control rats, both the number of submucosal ganglia and neurons decreased with age. However, in HF rats this happened earlier, as by p45 HF had ganglia and neuron counts similar to p60 control rats (see Figures 2A,B). The number of neurons per ganglion and neuronal body area remained unchanged throughout the experiment. Almost 100% of the submucosal neurons displayed positive signal for N200K, a marker for sensory fibers (not shown).

**FIGURE 2:**
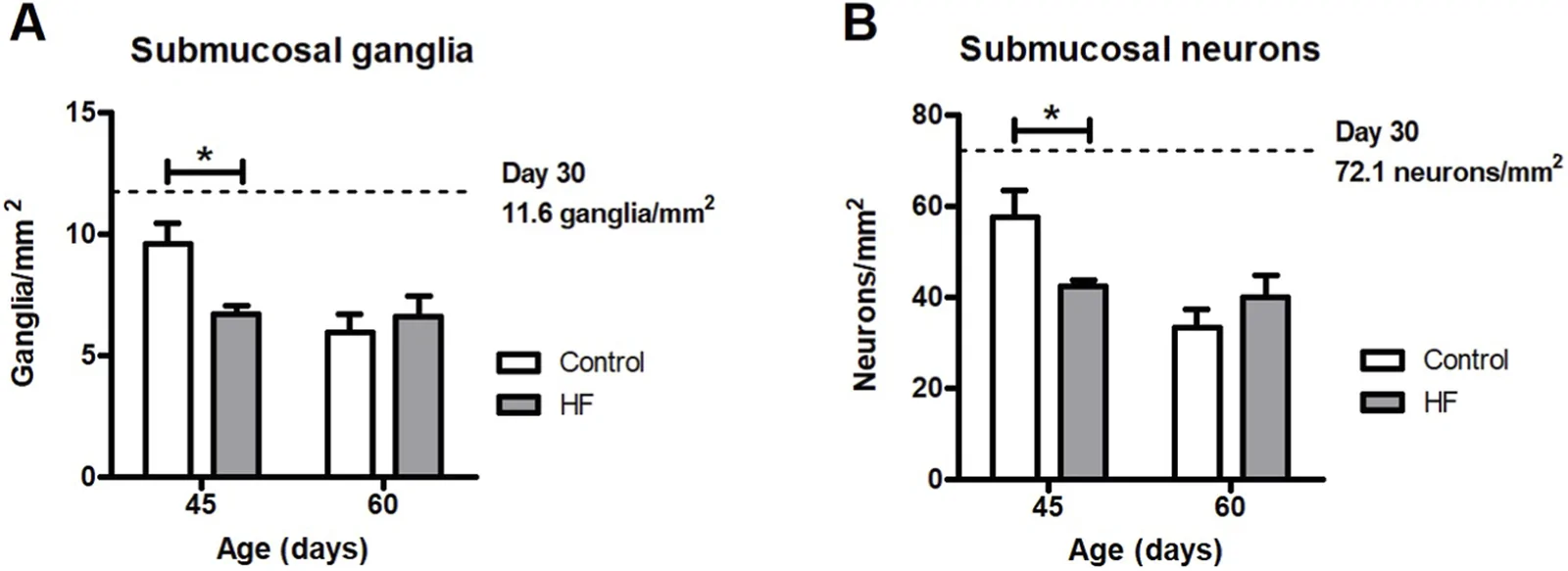
Morphological features in colon submucosal neurons from rats under a HF diet. **(A)** shows the number of ganglia and **(B)** the number of neurons per area. n = 4–5, *p < 0.05, two-way ANOVA, post-test Bonferroni.

The presence of four inflammation-associated neuropeptides was evaluated in colon submucosal neurons. The percentage of neurons that were positive for Somatostatin, Substance P, VIP and NPY was not modified by HF diet or age. Likewise, no differences were found when evaluating the somatic area in the neurons with the aforementioned phenotypes (Supplementary Figure S1).

Rats receiving an oral transplant of caecal microbiota obtained from HF animals (Cae group) did not show an increase in body or perigonadal mass weight, when compared to vehicle treated or heat-killed microbiota (HK) groups at the end of the 15 days treatment (see Figures 3A,B). However, the glycemia of Cae group was significantly higher to that of HK group (see Figure 3C). Notice that this cohort of animals was not subjected to overnight fasting before blood and tissue retrieval, and overall glycemia values are higher than those shown in Figure 1B. Notice that FMT was performed as proof of concept: we intended to transfer the HF diet-induced early phenotype by transferring the p60 HF rats caecal microbiota to younger recipients. Recipients were younger in order to perform readouts at the same age we had seen the earliest changes in HF diet-treated individuals (Figures 1, 2).

**FIGURE 3:**
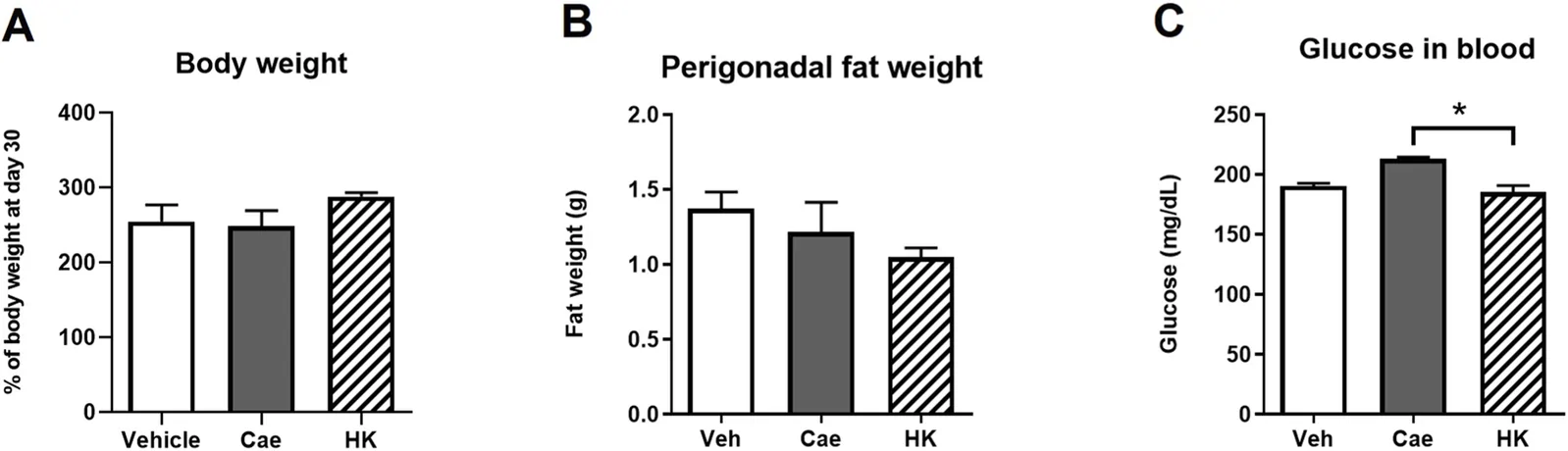
Anatomic and metabolic parameters in rats under caecal microbiota transplant. Body weight **(A)** was calculated as 100x((final weight–initial weight)/final weight). Vehicle (Veh) rats received saline orally from p30 to 45. Cae rats received caecal microbiota (taken at p60 from HF rats) daily from p30 to 45. HK rats received heat‐killed caecal microbiota under the same treatment scheme. Perigonadal fat weight **(B)** and blood glucose **(C)** were obtained to represent metabolic status. n = 3–5, *p < 0.05, one‐way ANOVA, post‐test Dunn's multiple comparisons test.

HF diet was associated with an early decrease in caecal microbiota richness, which became more evident with time (Supplementary Figure S2). The abundance of *Tanerellaceae* (Figure 4B) and *Bacteroidaceae* (Figure 4C) was increased early (p45) in HF diet rats. On the other hand, *Muribaculaceae* (Figure 4E) and *Lactobacillaceae* (Figure 4A) abundances were significantly lower in HF diet rats, but only at p60 and p90, respectively. The abundances of *Ruminococcaceae, Lachnospiraceae, Prevotellaceae, Rikenellaceae* and *Peptostreptococcaceae* (Figures 4D, F-I) were not changed by HF diet.

**FIGURE 4:**
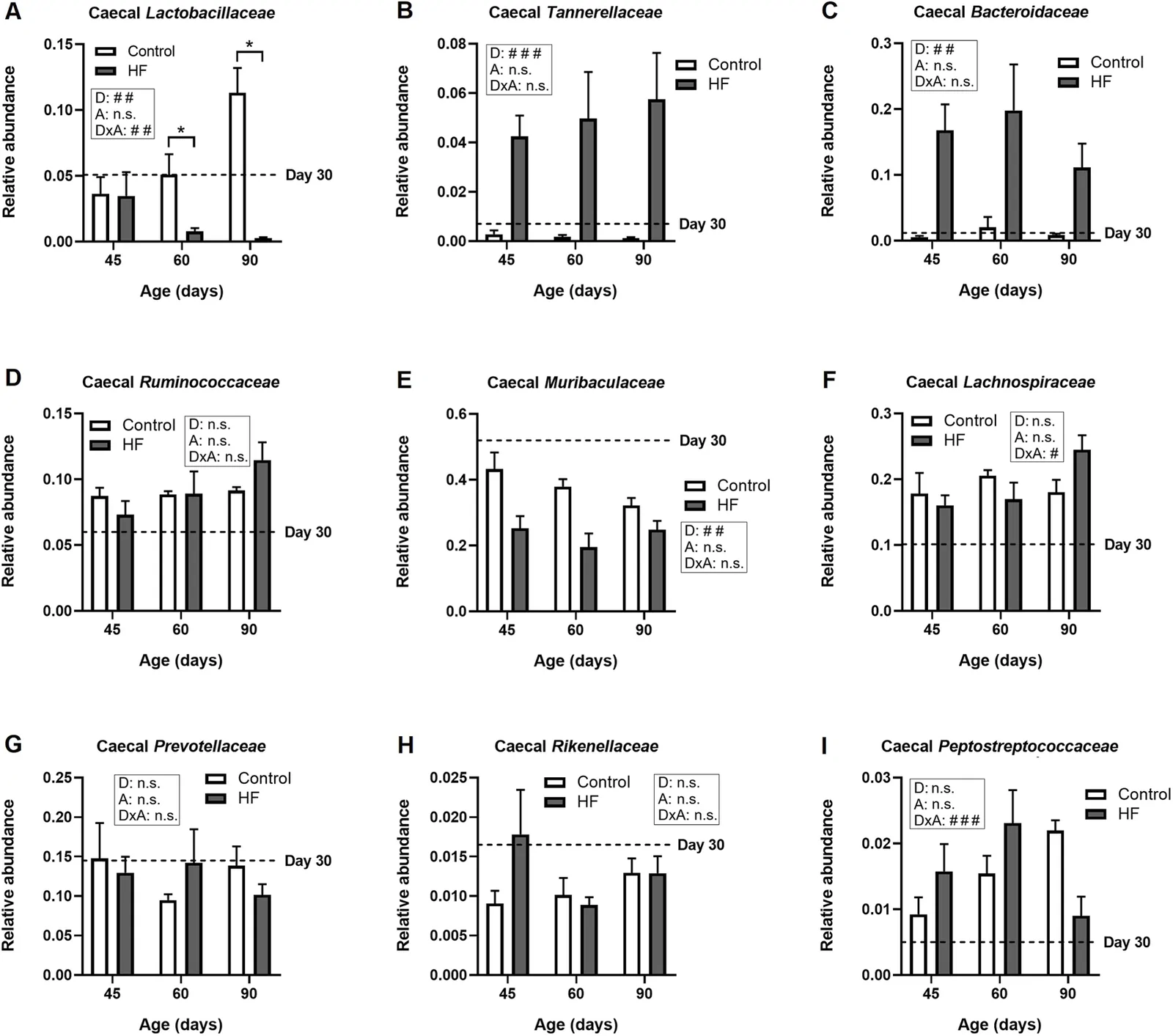
Changes in bacterial families from rat caecal microbiota under regular diet or HF diet. The following families are shown: *Lactobacillaceae* in **(A)**, *Tannerellaceae* in **(B)**, *Bacteroidaceae* in **(C)**, *Ruminococcaceae* in **(D)**, *Muribaculaceae* in **(E)**, *Lachnospiraceae* in **(F)**, *Prevotellaceae* in **(G)**, *Rikenellaceae* in **(H)** and *Peptostreptococcaceae* in **(I)**. The effects of diet **(D)**, age **(A)** and interaction (DxA) were established by two-way ANOVA and are shown with numerals: #p < 0.05, # #p < 0.01, # # #p < 0.001. The result of Bonferroni’s multiple comparison test is shown with asterisks: *p < 0.05. Disclosure: the data displayed in A was previously shown in a review by the same corresponding author (Julio-Pieper et al., 2021).

## 4 Discussion

Prophylactic efforts to decrease the occurrence of obesity during childhood and adolescence are paramount to improve the chances to a life without chronic disease. However, only 30% of obese adults have a history of obesity at a younger age (Simmonds et al., 2016). Therefore, measures such as body mass index (BMI) may not be totally reliable to predict future obesity. Our data obtained in a high‐fat diet-induced animal model of obesity shows that the submucosal plexus of the colonic enteric nervous system promptly reacts to this environmental change in young rats. This response, together with a modification in bacterial families in the large intestine, suggests that the gut may be a key piece of early metabolic shift, at least when unbalanced diet is the apparent culprit. This is not to say all members of a particular bacterial taxon will have convergent effects on their host’s metabolism; on the contrary, opposite effects have been found associated to different genera pertaining to the same family, and even on species under the same genus. For example, we show here that the abundance of Tannerellaceae family is greatly increased in the caecum of HF-diet rats (see Figure 4B) as early as 15 days after starting consuming the experimental diet. Within this family, *Parabacteroides* species abundance have been associated with increased thermogenesis of adipose tissue (Wu et al., 2019), while the genus has also been positively correlated with craving of obesogenic foods in rodents (de Wouters d'Oplinter et al., 2021). This highlights the need of performing a more refined description of gut microbial changes in juvenile preobese rats.

While the literature shows that many serum inflammatory markers are associated with obesity (Mindru et al., 2025), we were not able to detect differences in either pro or anti-inflammatory peptides in the enteric nervous system. We did find morphological changes in submucosal neurons; these arose before the HF-diet rats displayed increased adiposity or liver fat content. We may describe those as “ENS signatures” of pre-obesity in a similar fashion as others have talked about “microbiome signatures” of obesity onset in children (Rampelli et al., 2018; Nobrega et al., 2025). Although interesting, in the future this description should be complemented with its functional significance, i.e., changes in chemical code and/or plasticity.

Besides the advantage of identifying potential disease biomarkers, the present results highlight the value of microbiota transfer applied in a prophylactic manner. We were able to transplant one phenotypical aspect–i.e. increased glycaemia-from pre-obese rats to control recipients, and the opposite might possibly be true as well. It is important to state that we did not assess microbial engraftment in the recipient animals. Whether the transplanted microbiota successfully colonized is unknown, and the identity of taxa responsible for significant effects on the recipients is yet to be determined. Another caveat of our study is that we used caecal content as the source of microbiota. The caecum is a reliable sampling compartment in rodents, but potentially makes this result less translatable to a clinical setting, where faecal material is normally used. Also, characterization at the genus level should be prioritized. The possibility of providing with novel markers for obesity risk will improve the chances for successful interventions.

## Data Availability

Raw DNA sequences used in this investigation are publicly available in the NCBI SRA repository under BioProject accession number PRJNA1526311.

## Supplementary material

SUPPLEMENTARY MATERIAL FIGURE S1 Analysis of inflammation-associated neuropeptides in colon submucosal neurons from rats under a HF diet. **(A,D,G,J)** show the percentage of HuC/D neurons that were positive for Somatostatin, Substance P, VIP and NPY, respectively. **(B,E,H,K)** show the area of the soma for neurons from each respective phenotype. (C, F, I and L) represent the type of signal observed in each staining. n = 4–5, bar = 100 μm.

SUPPLEMENTARY MATERIAL FIGURE S2 Global analysis of rat caecal microbiota under regular or HF diet. A shows Alpha diversity, a measure of bacterial richness (i.e., the number of taxa) on each individual sample, whereas **(B)** illustrates the relative abundance for the most predominant bacterial families. The effects of diet (D), age **(A)** and interaction (DxA) were established by mixed-effect analysis and are shown with numerals: # #p < 0.01. The result of Bonferroni’s multiple comparison test is shown with asterisks: *p < 0.05. n = 4–5.
